# Gastric Adenocarcinoma Associated With CTLA-4 Haploinsufficiency in the Background of Common Variable Immunodeficiency: A Rare Case

**DOI:** 10.7759/cureus.112548

**Published:** 2026-07-12

**Authors:** Sara Tariq, Faiqa Tariq, Alan Lerman

**Affiliations:** 1 Internal Medicine, Guthrie Lourdes Hospital, Binghamton, USA; 2 Acute Medicine, Scunthorpe General Hospital, Scunthorpe, GBR; 3 Gastroenterology, Guthrie Lourdes Hospital, Binghamton, USA

**Keywords:** chronic gastritis, common variable immunodeficiency (cvid), ctla-4 haploinsufficiency, gastric adenocarcinoma, gastric intestinal metaplasia, pernicious anemia

## Abstract

Common variable immunodeficiency (CVID) is associated with immune dysregulation and an increased risk of gastric malignancy. The coexistence of CVID with autoimmune gastritis, pernicious anemia, and genetic defects such as cytotoxic T-lymphocyte antigen-4 (CTLA-4) haploinsufficiency may suggest a possible association with altered gastric adenocarcinoma risk. However, the clinical interplay remains incompletely understood and not well established. We present a case of a 54-year-old Caucasian woman with a high-grade intestinal tubulovillous adenoma in the stomach body, who developed gastric adenocarcinoma, which was subsequently resected, and had a recurrence of the tumor with a background of chronic lymphocytic gastritis almost four years later. She was later diagnosed with CVID and was found to have pernicious anemia and CTLA-4 haploinsufficiency. This case highlights the potential interplay between CVID, CTLA-4 haploinsufficiency, and autoimmune gastric pathology in the development of gastric adenocarcinoma. Early recurrence despite initial curative resection underscores the need for increased vigilance and consideration of structured surveillance strategies in high-risk patients with overlapping autoimmune gastric conditions. Further research is needed to guide screening and prevention in this population.

## Introduction

Gastric adenocarcinoma is emerging as a global health concern and is becoming one of the leading causes of cancer-related mortality. Among the most commonly diagnosed malignancies in patients with common variable immunodeficiency (CVID), gastric carcinoma is the second most common malignancy [1]. However, intestinal-type gastric adenocarcinoma was found to be more common in patients with CVID, and they usually have a background of chronic lymphocytic gastritis [2].

CVID constitutes a group of primary immunodeficiencies that impair antibody production and predispose to infections, autoimmune disorders, and malignancies. It is characterized by decreased levels of IgG, IgA, and IgM. Patients with CVID with hematologic or solid-organ malignancies, such as lymphoid malignancies (Hodgkin or non-Hodgkin lymphoma and mucosa-associated lymphoid tissue lymphoma) and gastric malignancies, are reported to have poor outcomes [3]. The risk factors for malignancy in patients with CVID remain under investigation. However, immune dysregulation, impaired tumor cell surveillance, proinflammatory cytokine release, and DNA damage have been found to contribute to the development of gastric adenocarcinoma [2,3]. It is diagnosed if the following criteria are fulfilled based on the European Society of Immunodeficiency: hypogammaglobulinemia, IgG/IgM or IgG/IgA (at least two standard deviations below the mean for age for IgG and one of the IgM or IgA isotypes); poor vaccine response and/or absence of isohemagglutinins; onset >4 years; and secondary causes excluded. The standard treatment is immunoglobulin replacement, administered intravenously (IVIG) or subcutaneously (SCIG), along with vaccinations, infection prevention, and management of autoimmune complications [4].

Pernicious anemia is a condition resulting from progressive atrophy of the gastric oxyntic mucosa, leading to impaired vitamin B12 absorption and achlorhydria. Its pathogenesis involves autoimmune dysfunction resulting in atrophy of the mucosal lining. Chronic inflammation due to autoimmune-mediated gastritis also increases the risk of gastric adenocarcinoma [5].

Heterogeneous pathogenic variants in CTLA-4 account for a minority of CVID cases [2]. CTLA-4 mutation carriers tend to develop gastric adenocarcinomas, but in this patient, genetic testing was performed later in the clinical course. She first presented with nonspecific symptoms of nausea and poor appetite and was diagnosed with intramucosal gastric adenocarcinoma. Additionally, she had underlying CVID, which appeared to be linked to the gastric tumor with other ongoing pathologies, including chronic lymphocytic gastritis and pernicious anemia. This case will highlight CTLA-4 haploinsufficiency as an emerging cause of immune dysregulation and increased risk of gastric adenocarcinoma.

## Case presentation

A 54-year-old Caucasian woman with a past medical history of lymphocytic microscopic colitis, adrenal insufficiency, and gastroesophageal reflux disease presented to the gastroenterology clinic with complaints of chronic diarrhea, decreased appetite, nausea, and weight loss. She had experienced chronic diarrhea for several years and was treated with budesonide, which provided only intermittent, limited relief. She denied vomiting, early satiety, and abdominal fullness. She had no history of chronic *Helicobacter pylori* (*H. pylori*) infection or a family history of gastric adenocarcinoma. Her father died of lung carcinoma at 35 years of age. She was a non-smoker and did not consume alcohol. Her vital signs were stable, and her body mass index was within the normal range. Physical examination revealed a soft, flat abdomen, normal bowel sounds, and no abdominal tenderness. She had undergone upper endoscopy approximately nine years earlier, which revealed no lesions or polyps and no evidence of celiac disease.

**Figure 1:**
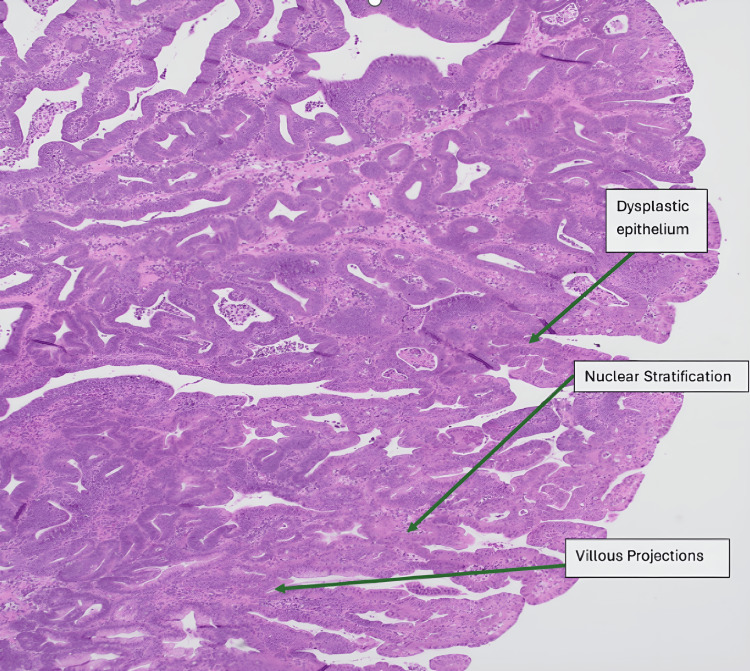
Low-power photomicrograph showing an adenoma with dysplastic epithelium, nuclear stratification, and villous projections (green arrows)

On day 0, upper endoscopy revealed a 10 cm polypoid mass extending along the posterior wall of the midgastric body. Biopsies demonstrated fragments of a tubulovillous adenoma with high-grade dysplasia, prominent acute gastritis, and negative *H. pylori* testing (Figure 1).

**Figure 2:**
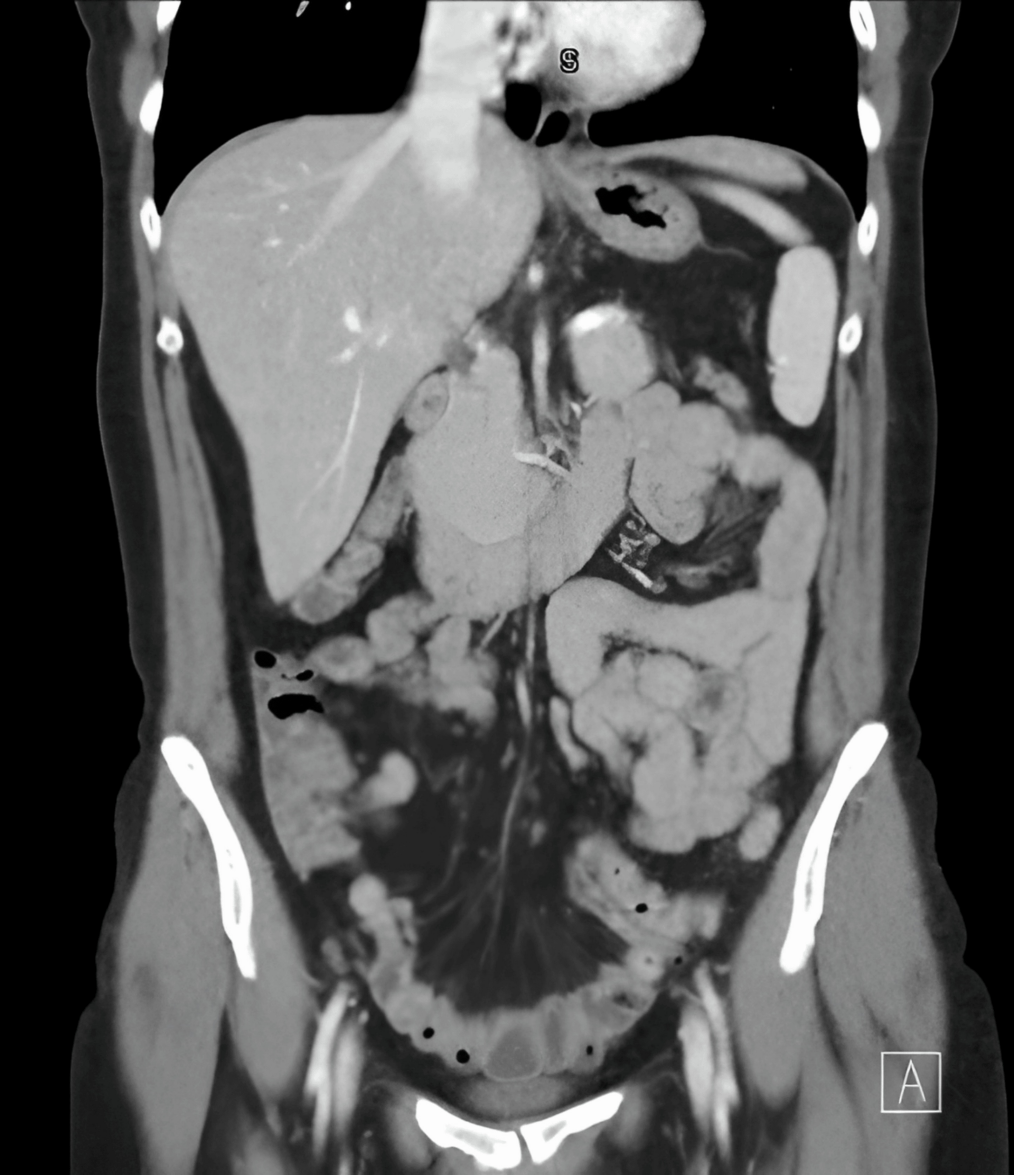
Coronal CT image of the abdomen and pelvis obtained without IV contrast CT: computed tomography, IV: intravenous

Eight weeks later, she underwent endoscopic mucosal resection of the gastric lesion. Pathologic examination revealed intramucosal adenocarcinoma (pT1a) arising in an intestinal-type tubulovillous adenoma. CT of the abdomen and pelvis performed to evaluate for metastatic disease was unremarkable (Figure 2).

**Figure 3:**
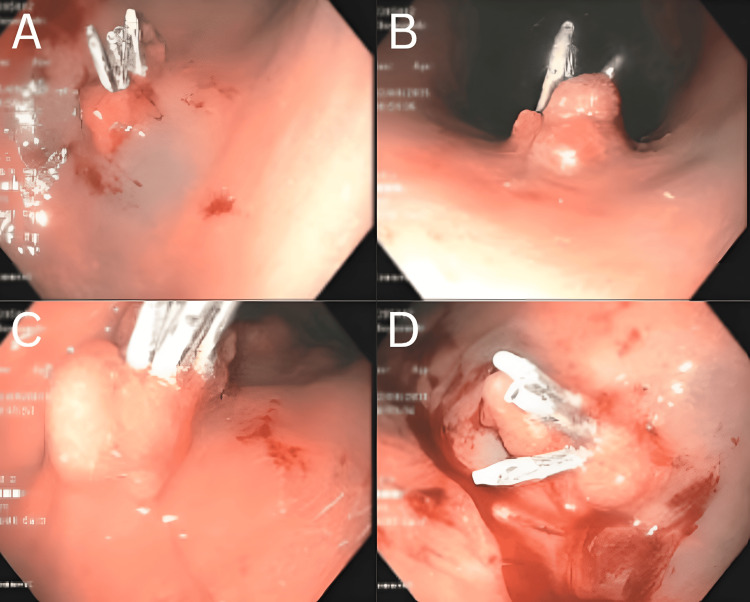
EGD revealing a gastric tumor on the greater curvature of the gastric body and the posterior wall of the stomach Panels A-D illustrate the sequential steps of gastric tumor resection performed during EGD. EGD: esophagogastroduodenoscopy

**Figure 4:**
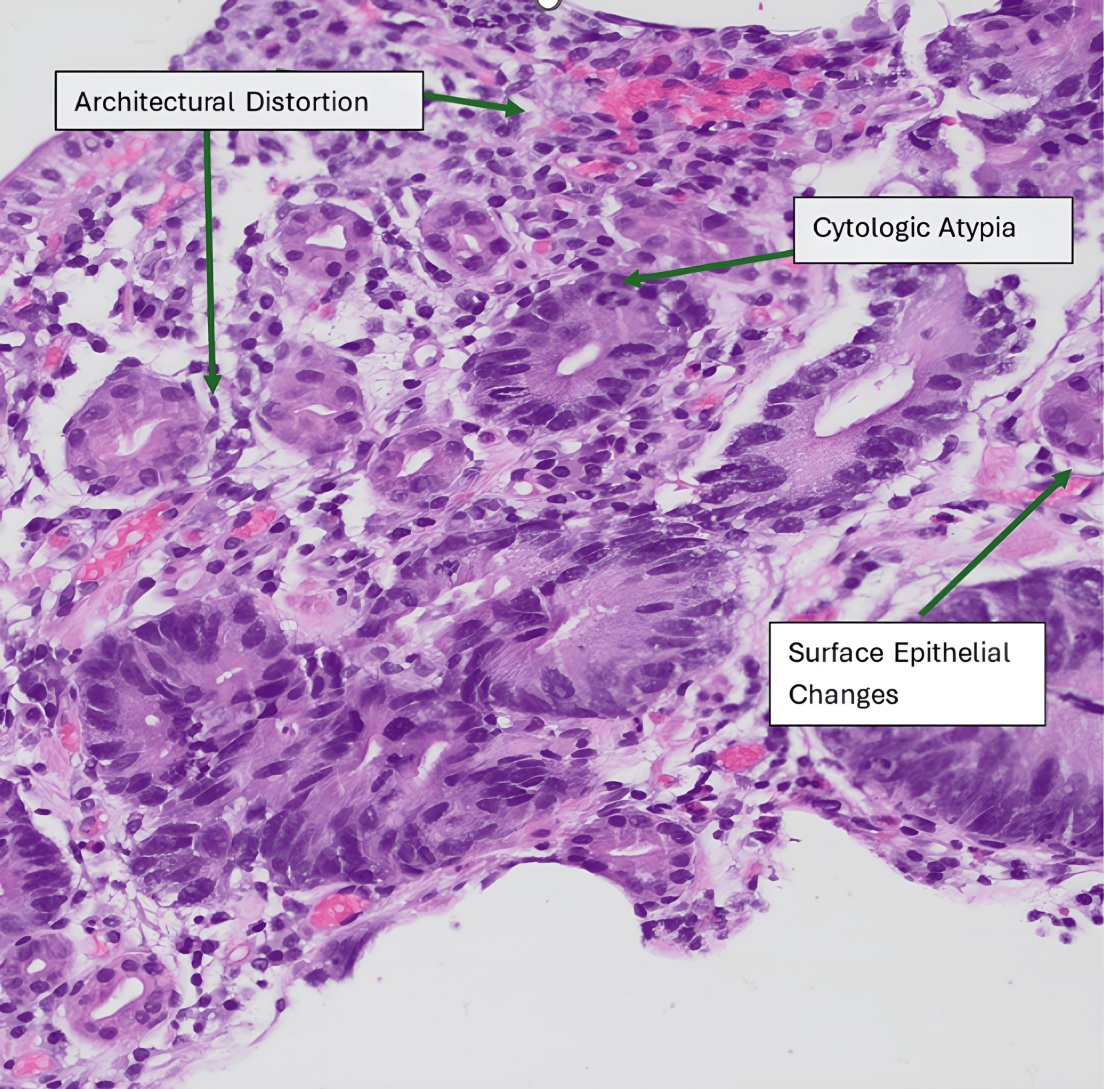
Histologic features of high-grade dysplasia, including architectural distortion, cytologic atypia, and surface epithelial changes (green arrows)

One month later, follow-up esophagogastroduodenoscopy (EGD) with biopsies demonstrated adenomatous glandular epithelium with at least high-grade dysplasia (Figures 3-4).

She subsequently underwent consultation with an interventional gastroenterologist for endoscopic ultrasound (EUS) to assess the feasibility of gastric resection and ultimately underwent repeat endoscopic mucosal resection rather than gastrectomy.

Pathologic examination of repeat EGD specimens after mucosal resection demonstrated fragments of tubular adenoma without residual adenocarcinoma, consistent with complete resection of the pT1a intramucosal adenocarcinoma. Because of the early-stage disease, no adjuvant therapy, including chemotherapy, was recommended. She subsequently underwent routine endoscopic surveillance.

Surveillance EGDs remained unremarkable for approximately four years, with no evidence of recurrence. During this surveillance period, she was diagnosed with pernicious anemia secondary to autoimmune gastritis (AIG) based on positive intrinsic factor antibodies and biopsy findings and was started on vitamin B12 injections.

**Figure 5:**
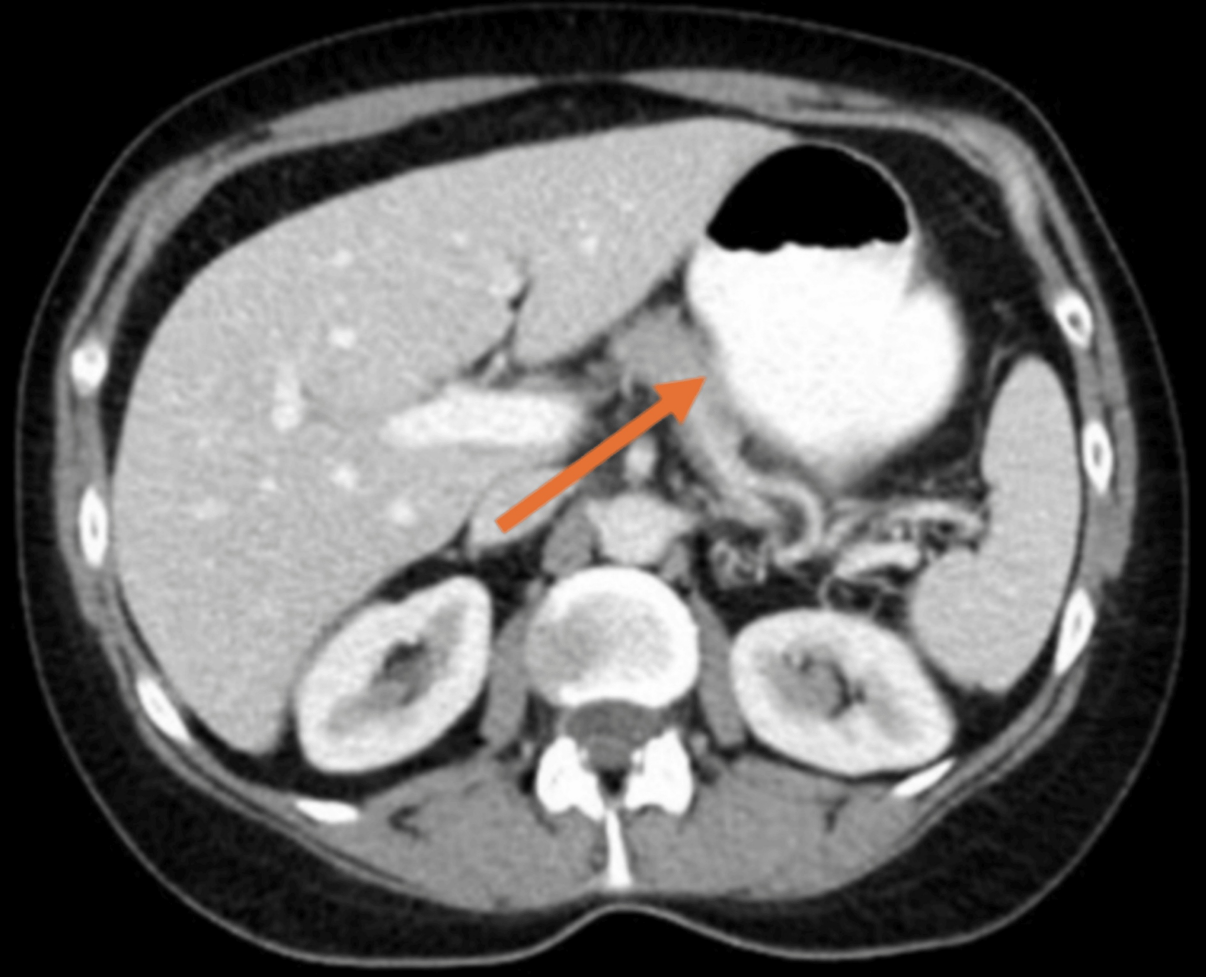
Contrast-enhanced CT of the abdomen and pelvis demonstrating an area of asymmetric mural thickening in the gastric antrum measuring 0.9 × 2.5 cm with possible reactive mesenteric lymph nodes CT: computed tomography

Approximately four years after the initial diagnosis, surveillance upper endoscopy demonstrated a well-differentiated intramucosal adenocarcinoma arising in a background of active chronic lymphocytic gastritis and intestinal metaplasia involving the gastric antrum. A second opinion at a tertiary care center confirmed the diagnosis. Follow-up contrast-enhanced CT of the abdomen and pelvis revealed asymmetric mural thickening of the antrum measuring 0.9 × 2.5 cm with possible reactive mesenteric lymph nodes (Figure 5).

She was referred to a tertiary cancer center for consideration of endoscopic resection and underwent evaluation, with EUS planned approximately one month later for staging.

Three months later, repeat endoscopy demonstrated a gastric antral fold and polyp with superficial moderately differentiated adenocarcinoma, along with active chronic gastritis involving the gastric body and fundus. *H. pylori* testing remained negative, and HER2, PMS2, and MLH1 expression were absent. Although endoscopic resection had been planned, a large ulcerated partially circumferential pyloric mass involving more than 50% of the circumference was identified, precluding endoscopic submucosal dissection (ESD) and making her a candidate for gastrectomy. Preoperative CT of the chest and PET demonstrated hypermetabolic diffuse gastric activity consistent with the known primary tumor, a few non-FDG-avid nonspecific pulmonary nodules, and no evidence of distant metastatic disease.

Approximately two months later, she underwent robotic subtotal gastrectomy with Billroth II reconstruction. Final pathology demonstrated pT1aN0 gastric adenocarcinoma with no metastatic involvement of 33 examined lymph nodes. She recovered well without postoperative complications or evidence of recurrence and continues to undergo surveillance EGDs.

During the same period, she underwent evaluation by an allergist/immunologist because of recurrent episodes of burning urticarial lesions involving the periorbital region and chest that occurred approximately once monthly and responded to cold compresses. An elimination diet produced temporary improvement in gastrointestinal symptoms; however, watery diarrhea persisted because of microscopic colitis despite budesonide therapy. She was subsequently diagnosed with CVID based on low serum IgG (467 mg/dL; reference range: 700-1600 mg/dL), IgM (9 mg/dL; reference range: 40-230 mg/dL), normal-low IgA (95 mg/dL; reference range: 70-400 mg/dL), a low B-cell count (9 cells/μL), a decreased CD4 T-cell count (179 cells/mm³), normal natural killer cell and granulocyte counts, and nonprotective pneumococcal vaccine antibody titers. A second opinion at a tertiary care center confirmed the diagnosis. Because of associated cytopenias, she was started on prophylactic trimethoprim-sulfamethoxazole (Bactrim) for infection prevention and later initiated IVIG therapy, which she tolerated well. Several months later, genetic testing identified inherited CTLA-4 haploinsufficiency (monoallelic pathogenic variant), and abatacept (125 mg subcutaneously once weekly) was added to her IVIG regimen.

The timeline of clinical events is summarized in Table 1.

**Table 1:** Timeline of clinical events EGD: esophagogastroduodenoscopy, EMR: endoscopic mucosal resection, CT: computed tomography, EUS: endoscopic ultrasound, pT1a: pathologic tumor stage 1a, CVID: common variable immunodeficiency, IgG: immunoglobulin G, IgM: immunoglobulin M, CD4: cluster of differentiation 4, IVIG: intravenous immunoglobulin, *H. pylori*: *Helicobacter pylori*, HER2: human epidermal growth factor receptor 2, MLH1: MutL Homolog 1, PMS2: postmeiotic segregation increased 2, ESD: endoscopic submucosal dissection, PET: positron emission tomography, FDG: fluorodeoxyglucose, pT1aN0: pathologic tumor stage 1a with no regional lymph node metastasis, CTLA-4: cytotoxic T-lymphocyte-associated protein 4. B12: vitamin B12, AIG: autoimmune gastritis

Time	Event/finding	Details
Approximately nine years before day 0	Prior endoscopies	No gastric lesions or polyps identified.
Day 0	EGD – initial discovery of gastric mass	A 10 cm polypoid mass was identified in the posterior wall of the mid-gastric body. Biopsy demonstrated a tubulovillous adenoma with high-grade dysplasia, acute gastritis, and negative *H. pylori* testing.
Eight weeks later	EMR	Pathology revealed intramucosal adenocarcinoma (pT1a) arising in an intestinal-type tubulovillous adenoma. CT of the abdomen and pelvis showed no evidence of metastatic disease.
Twelve weeks after day 0 (four weeks after EMR)	Follow-up EGD	Persistent adenomatous epithelium with at least high-grade dysplasia.
Approximately four months after day 0	Surgical consultation and EUS evaluation	Gastrectomy was considered; however, repeat EMR was performed instead.
Shortly thereafter	Repeat pathology	Tubular adenoma without residual adenocarcinoma, consistent with complete resection of the prior pT1a intramucosal carcinoma. No chemotherapy was recommended.
Approximately four years after day 0	Surveillance EGDs	No evidence of recurrence during surveillance.
Approximately four years after day 0	Surveillance EGD – recurrence	Well-differentiated intramucosal adenocarcinoma identified in the gastric antrum with active chronic lymphocytic gastritis and intestinal metaplasia. Diagnosis confirmed at a tertiary care center. CT of the abdomen and pelvis demonstrated asymmetric antral mural thickening (0.9 × 2.5 cm) with possible reactive mesenteric lymph nodes.
During the same surveillance period	Diagnosis of pernicious anemia	Diagnosed secondary to AIG and started on vitamin B12 injections.
Approximately three months later	Diagnosis of CVID	Laboratory evaluation showed low IgG and IgM levels, a low B-cell count, a decreased CD4 T-cell count, a normal natural killer cell count, and nonprotective pneumococcal antibody titers. Prophylactic trimethoprim-sulfamethoxazole (Bactrim) was initiated, followed by IVIG therapy.
Approximately one month later	Endoscopy before planned resection	Gastric antral fold/polyp demonstrated superficial moderately differentiated adenocarcinoma with active chronic gastritis. *H. pylori* remained negative; HER2 expression was absent, and MLH1 and PMS2 were absent. A large partially circumferential ulcerated pyloric mass precluded ESD, and gastrectomy was recommended.
Approximately two months later	Staging workup	CT of the chest and PET imaging demonstrated diffuse gastric uptake consistent with the known primary tumor, no distant metastases, and non-FDG-avid pulmonary nodules.
Approximately one month later	Robotic subtotal gastrectomy with Billroth II reconstruction	Final pathology demonstrated pT1aN0 gastric adenocarcinoma with no metastatic involvement of 33 lymph nodes.
Several months later	Genetic testing	CTLA-4 haploinsufficiency was identified, and abatacept was added to the ongoing IVIG regimen.
Ongoing follow-up	Continued management	The patient recovered well after gastrectomy without evidence of recurrence and continues surveillance EGD and management of CVID, AIG, and microscopic colitis.

## Discussion

CVID is known to predispose to infections, but recently, its association with gastric carcinoma has become increasingly recognized [6].

A prospective study of 220 patients with CVID, followed for 11 years, demonstrated a 47-fold increase in the risk of gastric adenocarcinoma [2,7]. Patients with CVID who were noted to have gastric adenocarcinoma had moderately to well-differentiated intestinal-type adenocarcinomas with severe atrophic pangastritis and an association of pernicious anemia in 10% of patients [2]. Although CVID is frequently associated with gastric adenocarcinoma, there are still no widely accepted screening guidelines for gastric adenocarcinoma in CVID due to its heterogeneous and variable presentations [8]. There is consensus on risk assessment in such patients, but whether IVIG treatment helps prevent gastric malignancies remains to be investigated.

A multicenter cross-sectional study in Spain found a prevalence of gastric adenocarcinoma of five (2.01%) among 250 patients with CVID. It demonstrated that cancer screening should be performed vigilantly in patients with CVID, identifying age, low CD4 cell count, increased IgM levels, immune dysregulation, and previous use of immunosuppressive drugs as potential predictors of neoplasia in patients with CVID [3].

Furthermore, the interlinked association among CVID, gastric adenocarcinoma, and lymphocytic gastritis has been demonstrated in a study by Petris et al. They evaluated 5793 patients with gastric adenocarcinoma. Six of them were reported to have CVID, and two of them had lymphocytic gastritis in the background. The gastric adenocarcinoma was of the intestinal type with a high number of intramural lymphocytes (ITLs). They highlighted the importance of considering CVID-associated gastric adenocarcinoma (CAGA) in a patient with gastritis and ITLs [9]. Similarly, lymphocytic gastritis was found in six (12.3%) of 30 patients with gastric adenocarcinoma, suggesting a role for immunologic dysfunction in its pathogenesis [10].

The genetic connection in patients with CVID and gastric adenocarcinoma was shown in a systematic review of 76 studies by Zheng et al., where they described that cytotoxic T-lymphocyte antigen-4 (CTLA-4) stands among the three most implicated genes responsible for DNA mismatch repair (others being ATM and CAR-MIL2) and gastric adenocarcinoma development in patients with CVID [11].

A study from 2018 reported a case of a 47-year-old female with CTLA-4 haploinsufficiency who developed gastric adenocarcinoma two years after her CTLA-4 haploinsufficiency diagnosis [12].

Pernicious anemia enhances the risk of gastric adenocarcinoma. A recent analysis from 2025 showed that AIG resulting in pernicious anemia through gradual atrophy of oxyntic mucosa can also be a risk factor for gastric adenocarcinoma. It also highlighted that AIG has been independently linked to gastric adenocarcinoma, and screening for gastric tumors in patients with AIG was recommended [13].

A 2018 study conducted in 455 Italian patients with CVID demonstrated a 10.1-fold increase in mortality in patients with CVID and gastric adenocarcinoma, suggesting the need for regular upper endoscopies in patients with CVID for diagnosing gastric adenocarcinoma early [14]. These findings are further supported by Van der Poorten et al., who proposed a novel screening protocol to detect early premalignant lesions and reduce the risk of gastric adenocarcinoma [15].

A summary of the above studies, including their objectives, key findings, and other associations, is presented in Table 2.

**Table 2:** Summary of relevant studies CVID: common variable immunodeficiency, CAGA: CVID-associated gastritis, ITLs: intramural lymphocytes, GI: gastrointestinal, PI: primary immunodeficiency, CTLA-4: cytotoxic T-lymphocyte-associated protein 4, N/A: not applicable

Study design	Hypothesis/objective	Key findings	Other associations/risk factors
Prospective (1985, Kinlen et al.) [7]	220 patients with CVID out of 377 primary hypogammaglobulinemia patients were followed for 11 years to study the incidence of cancer	Fivefold increased risk of cancer, 47-fold gastric adenocarcinoma, 30-fold-lymphomas, gastric adenocarcinoma, intestinal type adenocarcinomas, moderately to well-differentiated	Atrophic pan gastritis, pernicious anemia (10% of patients)
Multicenter cross-sectional study (2024, Cabañero-Navalon et al.) [3]	250 patients with CVID were studied to characterize the clinical profile of cancer in patients with CVID and to identify independent risk factors associated with malignancy development, focusing on the role of immune dysregulation	2% increased gastric adenocarcinoma prevalence, higher rates of immune dysregulation in cancer development (81.58%, p = 0.01)	N/A
Meta-analysis (2014, Petris et al.) [9]	5793 patients with gastric adenocarcinoma were studied to determine the morphological features of CAGA and of the background gastritis	Six patients with CVID had gastric adenocarcinoma, intestinal type adenocarcinomas, high number of ITLs	Severe atrophic metaplastic pangastritis with extensive dysplasia was present in the background in four cases, features of lymphocytic gastritis present in two cases
Case series (1994, Griffiths et al.) [10]	A series of primary gastric lymphomas and adenocarcinomas was reviewed to assess the prevalence of lymphocytic gastritis	Lymphocytic gastritis was more prevalent in patients with gastric adenocarcinoma (16 of 130 cases; 12.3%)	N/A
Systematic review (2022, Zheng et al.) [11]	76 publications were reviewed to study the association of primary immunodeficiencies with a potential risk factor for early-onset GI cancer	149 patients with PI and GI cancer, 95 developed gastric adenocarcinoma, CTLA-4 among the top three genes for gastric adenocarcinoma development in patients with CVID	CVID most common PI associated with GI cancer
Cohort study (2018, Egg et al.) [12]	Clinical manifestations and details of the clinical history were assessed in a worldwide cohort of 184 CTLA4 mutation carriers with documentation of malignancies	184 CTLA-4 mutation carriers, 17 malignancies were documented, five reported to be gastric adenocarcinoma	N/A
Prospective study (2018, Pulvirenti et al.) [14]	455 Italian patients with CVID were assessed for risks and mortality of cancers	455 Italian patients with CVID had a 10.1-fold increased mortality due to gastric adenocarcinoma	N/A

This case represents a patient who was diagnosed with intramucosal gastric adenocarcinoma. She was also investigated for CVID due to having multiple recurrent infections and food intolerance and was found to have pernicious anemia as well as chronic lymphocytic gastritis. Later, her genetic testing revealed CTLA-4 haploinsufficiency. Based on the sequence of events, a possible association between CTLA-4 haploinsufficiency and increased risk of gastric adenocarcinoma in patients with CVID cannot be excluded.

## Conclusions

Our case demonstrates that clinicians should maintain a high index of suspicion for gastric adenocarcinoma in patients with CVID, especially if they have concomitant pernicious anemia or chronic lymphocytic gastritis. Therefore, it is recommended that a proper screening protocol be established for intestinal-type gastric adenocarcinoma in patients with CVID and CTLA-4 haploinsufficiency, especially in the context of chronic lymphocytic gastritis or pernicious anemia. Additionally, the role of immunoglobulin replacement therapy and whether it and abatacept reduce the risk of gastric adenocarcinoma in patients with CVID should be investigated in larger studies to improve health outcomes.
